# Supporting Women after Obstetric Fistula Surgery to Enhance Their Social Participation and Inclusion

**DOI:** 10.3390/ijerph21091201

**Published:** 2024-09-10

**Authors:** Tibeb Debele, Heather M. Aldersey, Danielle Macdonald, Zelalem Mengistu, Dawit Gebeyehu Mekonnen, Beata Batorowicz

**Affiliations:** 1 Department of Rehabilitation Science, School of Rehabilitation Therapy, Queen’s University, 31 George St., Kingston, ON K7L 3N6, Canada; hma@queensu.ca (H.M.A.); 19dgm2@queensu.ca (D.G.M.); beata.batorowicz@queensu.ca (B.B.); 2 Department of Clinical Midwifery, School of Midwifery, College of Medicine and Health Sciences, University of Gondar, Gondar 196, Ethiopia; 3 School of Nursing, Queen’s University, Kingston, ON K7L 3N6, Canada; danielle.macdonald@queensu.ca; 4 Department of Obstetrics and Gynecology, University of Global Health Equity, Kigali 6955, Rwanda; zmengistu@ughe.org

**Keywords:** obstetric fistula, obstetric complication, formal support, informal support, social inclusion, participation, Ethiopia, Africa

## Abstract

Obstetric fistula is a childbirth complication causing abnormal openings between the urinary, bowel, and genital tracts, leading to involuntary leakage and potential long-term disability. Even after surgical repair, women continue to face psychological and social challenges that affect their social inclusion and participation. This study explored family and service provider perspectives on current support systems and identified gaps affecting women’s inclusion and participation post-fistula surgery. Building on a prior study of women who underwent obstetric fistula surgical repair, we qualitatively examined available formal and informal post-surgical supports in Ethiopia. We conducted 20 interviews with family members and service providers and analyzed them using Charmaz’s grounded theory inductive analysis approach. We identified four themes that indicated the available formal support in fistula care, the impact of formal support on women’s social participation and inclusion, the gaps in formal support systems, and post-surgery informal supports and their challenges. Both groups believed support needs for women after surgery remain unmet, highlighting the need to strengthen holistic support services to improve women’s social inclusion and participation. This study contributes to limited research on formal and informal support for women, emphasizing the need for enhanced economic, psychological, and sexual health-related support post-obstetric fistula surgery.

## 1. Introduction

Obstetric fistula (OF) is a birth-related complication characterized by abnormal openings in the genital tract, urinary tract, or rectum, leading to involuntary leakage of bodily excretion and a potential long-term disability [1,2]. While OF has been eradicated in high-income countries, it remains prevalent in low- and middle-income countries (LMICs), particularly in sub-Saharan Africa, due to barriers in accessing quality maternal healthcare [3,4,5]. In addition, in recent years, there has been a rise in OF cases from iatrogenic injuries during operative deliveries [6,7]. The World Health Organization (WHO) reports 50,000 to 100,000 new cases of OF annually in a global context, with Ethiopia alone experiencing an estimated 3000 to 7000 new cases every year [8,9].

The treatment for OF involves surgical repair of the abnormal communication in various organs that resulted during labor. In Ethiopia, various health institutions provide surgical services for women, including the Hamlin Fistula Centers and some university hospitals. Private nonprofit Hamlin Fistula Centers are located in the capital city, Addis Ababa, with another five centers in various regions of the country. The Addis Ababa Center is the leading fistula center in the world. It has a rehabilitation center called Desta Mender, where fistula patients are provided with counseling, literacy, numeracy classes, and vocational and life skills training [10]. The university hospitals include the University of Gondar Specialized Hospital, Jimma University, and Arsi University hospitals, which provide fistula repair services [11]. Also, a Non-Governmental Organization (NGO) called Healing Hands of Joy (HHJ) works in four regions of the country and empowers women recovering from fistula through education, economic support, and counseling to transform their lives and aid in their return to the community [12].

While progress has been made in providing surgical treatment for women affected by OF, studies have shown that physical improvement alone may not be enough to address the continued psychological and social challenges that women experience [13,14,15,16,17]. Prior to undergoing surgical repair, individuals may encounter psychosocial difficulties stemming from urinary and fecal incontinence, as well as associated health issues and the emotional toll of stillbirth, which can lead to stigma, discrimination, depression, anxiety, and thoughts of self-harm [18,19,20]. Even after receiving surgical intervention, women may still face medical and social obstacles such as persistent infertility, changes in their marital roles, and a potential impact on future reproductive capabilities [21]. Such challenges also have the potential to limit women’s social participation and inclusion, which highlights the need for a coordinated approach to tackling the issue [22,23]. Therefore, it is imperative to recognize that women affected by OF may require a spectrum of support to overcome adversity and return to their families and communities.

The role of support, such as enhancing well-being and resilience during challenging times [24] and facilitating social inclusion and participation [25,26] has been documented in the broader literature. According to Lipman and Longino, support can be divided into informal and formal based on its source [27]. Informal support, also called natural support or social support, encompasses assistance offered by family and friends as a combination of emotional, practical, and financial assistance [28,29]. On the other hand, formal support is provided by service providers in a more structured way [28,30,31,32].

Despite the important role of formal and informal support, little research is available about either form of support for women post-OF surgery. Researchers found that even though familial support is deemed one of the crucial supports for the return of women to society after OF surgery, many women lack this support system [33,34]. A few studies conducted in Ethiopia indicated that while women receive limited formal and informal support, they still have unmet needs regarding employment, reproductive health, family, and marital life [21,35. An ethnographic study from Ghana showed that women with OF perceive support in terms of tangible assistance focused on access to information and finance [36]. However, there is a scarcity of research regarding the available formal and informal supports that facilitate, contribute to, or impact women’s social participation and inclusion experiences after OF surgery. Therefore, this study aimed to explore the perspectives of family members and service providers regarding currently available formal and informal support for women, how such support could enhance women’s social inclusion, and identify what is missing from this support system. Including these views will enable the co-design of the best services for women.

## 2. Materials and Methods

### 2.1. Study Design

We used Charmaz’s grounded theory approach [37,38] to conduct a qualitative exploration of the formal and informal supports available to women who had OF surgery. In a previous study, we explored women’s perspectives on the topic [21]. The current study forms part of a grounded theory study that aimed at developing a substantive theory regarding the social inclusion process of women post-OF surgery in Ethiopia.

### 2.2. Settings

This institutional and community-based study comprised two participant groups: family members of women with OF and service providers. Family members were recruited from the Amhara region, Ethiopia, and service providers were recruited from two fistula institutions—The University of Gondar Generalized Teaching Hospital, and the Addis Ababa Hamlin Fistula Center—and a non-governmental organization, HHJ.

### 2.3. Study Population and Sampling

Twenty individuals participated in this study: nine family members and 11 service providers. Family members were identified through women with OF previously interviewed for a grounded theory study on the social inclusion process of women post-surgical treatment. Verbal consent was obtained from these women to contact their family members, who were then invited to participate in this study by the lead author (T.D.). Selected families chose interview locations and received information letters and consent forms beforehand. Eligible family members had a marital, parent–child, or extended family relationship with the woman, cohabited with her, and were at least 18 years old. The sample reflected diverse family relationships, such as child, parent, and spouse of the woman with OF.

We recruited service providers involved in caring for women with OF using purposive sampling and maximum variation criteria like years of service, profession, and gender. Participants aged 18 or above who had worked at fistula institutions for at least six months were recruited through poster calls or personal communication. Written informed consent was obtained prior to the interviews. Participants who were unable to write provided a fingerprint signature, which was witnessed by the researcher after the information letter and consent form were read to them and they were given the opportunity to ask any questions that needed clarification. After each interview, participants were reimbursed an amount of CAD 10 for their time. Table 1 shows the characteristics of the study participants.

### 2.4. Data Collection

We used semi-structured interview guides developed by a team of five researchers, including content experts in rehabilitation sciences, social participation, qualitative research, and maternal and child health. Questions were based on the ICF framework [39] and aimed to understand health and disability from the perspectives of an individual’s life situation as well as other contextual factors [40]. These factors include the physical, social, and attitudinal environment in which individuals live and function, which can act as barriers or facilitators to participation. In our study, these factors were assessed through family members and healthcare providers. Family members were interviewed face-to-face at preferred locations like their homes or hospitals, with each interview lasting 30 to 70 min. Service providers were interviewed in person and virtually via Zoom (version 6.0.11, Queen’s University, Kingston, ON, Canada), with each interview lasting 60 to 90 min.

All in-person interviews with service providers were conducted in the participants’ offices. Interview guides were prepared in English and translated into Amharic (the local language). All interviews were conducted in Amharic.

### 2.5. Data Analysis

Charmaz’s inductive data analysis approach was employed to analyze the data [38]. All interviews were audio recorded except for one professional who refused audio recording. For the participant who declined the recording, the primary author (T.D.) took extensive notes while conducting the interview [41]. All audio recordings were transcribed verbatim, and T.D. read the transcripts multiple times to understand the data comprehensively [42]. Next, T.D. and a second coder (D.G.), who are native speakers of Amharic, did line-by-line coding in Amharic to produce inductive ideas and selected the most significant ideas through focused coding [38,43]. A professional translator translated the focused codes to English to communicate with the senior author (B.B.) and continue the coding process. Finally, all authors developed sub-themes and themes and identified supporting exemplars.

We employed multiple strategies to ensure this study’s rigor and trustworthiness, including building rapport and extensively engaging with the study participants before collecting data. The first author maintained a reflexive journal and wrote memos throughout this study [44]. To ensure accuracy, we used triangulation by involving at least two coders in the entire coding process [45]. We conducted member checking with the professional who declined audio recording during the interview. We presented the notes taken during the interview to the professional and sought their feedback to improve the accuracy and completeness of the recorded information [46,47].

## 3. Results

We identified four significant themes. The first three themes focused on available supports in fistula care, their impact on women’s social participation, and observed gaps in formal support systems. These themes were consistent across interviews from both service providers and family members, indicating a shared understanding of existing support structures’ importance and limitations. The fourth theme reflected the role of family as a support system during challenging times, offering insights into available post-surgery informal supports for women with obstetric fistula, their impact on social inclusion, and associated challenges (see Table 2).

### 3.1. Theme One: Holistic Support Provision

The theme of holistic support provision highlights holistic support services for women at fistula treatment facilities and from HHJ that aim to empower women and improve their well-being. This theme has two sub-themes: (I) Vocational and educational opportunities and (II) Spiritual, psychological, and physical support.

#### 3.1.1. Vocational and Educational Opportunities

Service providers and family members noted that fistula institutions and HHJ offer educational and tailored vocational and support programs post-surgery, underscoring the importance of empowering women to pursue their future goals through personalized micro-business plans. One service provider stated:

“Women who go to the HHJ are given money. They are asked what they want to do. Women may say opening a shop, rearing a sheep, raising chicken, beekeeping, or a farm if she wants, she chooses herself, HHJ gives her the money, and she works.”

(P-P-06)

Another service provider addressed how women make their own business plans, and the fistula institutions mainly provide monetary support and connect women to other organizations that can provide hands-on practice:

“Women will choose their business plan and communicate with women’s affairs. This means they started claiming their rights…And the fistula institutions will deposit money into their account…and contact women’s affairs to give on-the-job support.”

(P-P-04)

Another service provider discussed how they track the progress of women’s vocations once they receive initial support:

“We [HHJ] followed women we supported to see if the support was successful and how their lives had changed. We follow up with other stakeholders in the district to see if there are changes in their lives, especially after we started providing the IGA (Income Generating Support) support.”

(P-P-10)

The other topic discussed by service providers was the role of the fistula institutions and the HHJ in enhancing the education and skill acquisition of women post-OF surgery. A public health professional highlighted how women who drop out of school are supported to return to school by providing essential educational materials:

“Those who are eligible for education, that is, those who are looking for education and have been affected by this problem [fistula] because they were made drop out of school and get married, or those mothers who want to start education now, we identify them and provide educational support to return to school such as a notebook, pens, school uniforms or any payments related to education. These supports are among the things that empower and help women reintegrate.”

(P-P-10)

Another service provider described how women awaiting surgery were engaged in skill development activities, preparing them to establish a vocation upon leaving the fistula institutions. “They [women] learn handicrafts such as crochet. A woman is assigned to teach them embroidery, and they do that for three months [until they receive surgery]” (P-P-01). In addition to the empowerment activities through educational and vocational services, women were also provided with well-being services such as spiritual, psychological, and physical support.

#### 3.1.2. Spiritual, Psychological, and Physical Support

The sub-theme of spiritual, psychological, and physical support reflects the rehabilitation support for women aimed at addressing their multidimensional well-being. The participants reported delivering and receiving spiritual, psychological, emotional, and physical support.

Service providers and family members discussed how women affected by OF are on a continuous spiritual quest to understand why OF happened to them. Also, many women were excluded from various spiritual activities. To address these challenges, fistula institutions offered spiritual therapy. Also, service providers connect religious leaders with women and organize religious services for those who need them. One service provider said:

“For example, if a woman wants holy water and believes it cures her, we send her to a nearby holy water to get baptism…if we confirm it is not harmful, we support them with whatever [spiritual services] they may need. We link them to [spiritual fathers] and to get counseling…[women] might bring infants who are not baptized. So, when they bring such infants, we support them in getting the baptism services [at the church] and coming back here [to the facility] to celebrate the ceremony just like they would at their homes.”

(P-P-10)

Family members also stressed the significance of spiritual counseling for women. One mother noted how the spiritual counseling her daughter received greatly improved her daughter’s psychological well-being:

“…she [daughter] told me a priest provided her a counseling service. She told me, ‘I will be forever satisfied by his counseling’ …Her prior and current thoughts are completely different. I can say she is happy now.”

(P-F-07)

Service providers and family members also talked about the psychological services and support offered to women after OF surgery. According to one service provider, this psychological support significantly contributed to women’s social inclusion process:

“…they have lost their marriages, children, and families because of the fistula; they have lost community. So, one mother might come carrying five to six problems. If you treat only the physical illness of a mother who carries five or six problems or who came from an uneducated family, she will be lost on the road; that is not right because the service would be incomplete. So, we are working on a broad counseling activity [addressing] this problem could happen to anyone, and she might regain her health and return to her previous identity.”

(P-P-11)

Another service provider said:

“After the surgical repair, we gave appointments to the woman weekly to see if she was coping. What has happened in the past is this thing [fistula]. In the future, we work on what she can do. We tell her she can do things, have a child, and get married.”

(P-P-07)

Likewise, one husband mentioned how his wife received crucial psychological counseling services at the fistula center following her surgical treatment, which he deemed the most important care:

“She used to receive much counseling; they told her to stay strong. After she was [discharged], she came once a week, and they counseled her. That is the most crucial thing that benefited her. The counseling benefited me as well.”

(P-F-01)

Alongside psychological support, service providers emphasized physical recovery support for women post-surgery. The participants noted mobility challenges, particularly for those women who were immobile after childbirth. One service provider highlighted how pelvic floor exercises could alleviate urinary leakage and enable participation in daily activities and social interactions without limitations after surgical correction:

“The doctor does an operation on the hole, and after the hole is repaired, she might leak urine when she coughs, sneezes, lifts heavy loads, or when she gets older. So, to prevent this [leakage], we will teach her to do pelvic floor exercises.”

(P-P-05)

A husband appreciated the physical support his wife received at the fistula institution, noting how these experiences became part of her routine even after returning home.

During the discussion, a service provider shared insights about facilitating alternative physical treatments for women with unsuccessful fistula surgery. They elaborated on supplementary surgical procedures designed to enhance overall well-being post-surgery:

“Sometimes, when their bladder is severely damaged or after the fistula is repaired, there is stress, which means when the urethra is damaged, it starts to leak. Even if there is no hole, they are not still dry. That further affects their quality of life. For these kinds of patients, we do diversion surgery…so the urine comes through the abdomen, which is permanent.”

(P-P-03)

### 3.2. Targeted Interventions and Supports to Enhance Social Participation

The theme targeted interventions and supports to enhance social participation represents the perspectives of family members and service providers, focusing on how services at the fistula institutions and HHJ aimed to enhance women’s social participation. Sub-themes include rebuilding connections with peers and family and nurturing dignity through family-like care.

#### 3.2.1. Rebuilding Connections with Peers and Family

The sub-theme of rebuilding connections with peers and family reflects how the support in the fistula institutes and the HHJ aimed to facilitate the social relationships of women who have been once affected by their fistula experiences. Service providers and family members discussed that women received formal support at the institutions, enabling them to establish new peer support groups and to link them with their family members.

Service providers noted how group therapy facilitates informal communication and strengthens social bonds for women. One service provider said, “We have group therapy one day a week; we want them to feel they are at their homes. We will make coffee, eat kolo [cultural snack], and have a get-together” (P-P-11).

Another service provider discussed how these experiences of meeting new people made women feel they were socially accepted:

“…What is the good thing is that women will meet their colleagues when they come here [fistula treatment facility] because they meet people with similar health conditions and feel free to share their experiences. So they feel more socially sound.”

(P-P-09)

Furthermore, a service provider emphasized their efforts to promote peer support at fistula institutions to facilitate long-term communication and exchange experiences among women:

“When I talk to them [fistula patients], they might feel I am trying to convince them to cope. However, when I present them with an example of someone who has passed through such a problem [fistula], they believe them. They believe them very much. Therefore, we help them exchange contact numbers.”

(P-P-08)

One family member who stayed with their loved one during treatment noted that women in the fistula facilities shared similar experiences, which helped them overcome shame and become more open. She also spoke of the positive impact of peer support both for herself and their family member and its contribution to their return home:

“…at that time [time of admission], because they [fistula patients] were uniform with a similar problem, women were not ashamed or hiding anything. Therefore, we [my family member with OF and I] felt nothing when we returned to the community. We did not feel anything or show any embarrassment.”

(P-F-09)

Service providers not only offered peer support but also put efforts into helping women reconnect with their loved ones, especially their husbands, after surgery at fistula centers. One provider discussed a reintegration support program that aimed at rebuilding relationships with loved ones and community members. One service provider discussed the role of such initiatives in reconnecting women with their spouses and said:

“If the woman is separated from her husband and if he is not present in a male family workshop during the reintegration support, we go together [with women to her community] and bring them [husband and wife] closer and help them negotiate…last time we did various activities to reunite around 30 mothers to go back to their previous marriage.”

(P-P-10)

One husband expressed appreciation for the services tailored to male family members, mentioning how he received counseling on staying with his wife and the support he could provide her. “They counseled me not to leave our children and that she [wife] should not suffer in the house from now on, also that she might experience complications if she gives birth again. They told me all these things” (P-F-03).

#### 3.2.2. Nurturing Dignity through Family-like Care

The sub-theme of nurturing dignity through family-like care reflects the services given to women at the fistula institutions and the HHJ. Both service providers and family members discussed how women received positive and supportive healthcare services that respect their dignity.

Service providers discussed the supportive approach of the rehabilitation staff towards women, underscoring their role in addressing women’s psychological well-being. “With the staff’s support, we do extensive work to make their [women] rehabilitation good so they can be psychologically well-treated and discharged [back home].”

(P-P-10)

Service providers highlighted the isolating experiences women faced and offered care and support to help them overcome difficulties, with one nurse stressing the importance of friendly care for women’s mental health.

“I have to care for them [fistula patients] until they are discharged from the hospital. I am responsible for caring for them; the psychological care I provide is critical. I change the draw sheet frequently. I mean, even their family isolates them, and they are embarrassed. We [service providers] approach them friendly. So, they do not even call us nurses; they call us by [by our names]. We care for them.”

(P-P-01)

Another service provider supported the above claim and discussed how women are treated like family members, and the familial connections continue once women leave the fistula institutions.

“Fistula patients are very much like a family. I used to bring them my children’s clothes because they [women] might have children with them. Even this can create a bond. Because we are women, we have sympathy; some of us have kids, and we think about what if this problem occurred to one of us…because of this, we think about each other like sisters. They even call us after they go home.”

(P-P-08)

Family members also expressed their appreciation for the positive and cooperative attitudes of the staff working at the fistula institutions and the HHJ. One family member said: “We had a family-like love in the [fistula institutes], just like one family, all the patients, nurses, and doctors. We cried when we separated [at discharge]. It was a fantastic experience.” (P-F-09).

One husband discussed the support his wife received from the service providers and how it positively affected her social inclusion experiences, saying:

“The support received is good in that, after she had her surgery, she perceived people saw her in a negative way, so this support [from the service providers] enables her to understand there are many people around her and that she will not face the same problem in the future because she has people who support her, this also enables her to forget about the occurrence of the fistula, and work anything and live like any person.”

(P-F-04)

Both service providers and family members recognized the availability of services that aim to build women’s connections with others and dignified care that enables women to engage socially.

### 3.3. Unveiling Gaps in the Formal Support System

This theme, unveiling gaps in the formal support system, discusses areas where formal support for women may be inadequate, and has two sub-themes: insufficient and fragmented services and the need for strengthened economic and counseling support.

#### 3.3.1. Insufficient and Fragmented Services

In the sub-theme insufficient and fragmented services, the participants identified gaps in formal support for women post-OF surgery, including an insufficient emphasis on rehabilitation services beyond surgical repair, discontinuity in formal care during the transition to society, and the absence of a sustainable support system.

Service providers discussed how they aimed to provide comprehensive services but raised concerns regarding insufficient support centers for women. One service provider indicated the need to expand the rehabilitation services beyond the fistula centers:

“We need to add more rehabilitation services. They [women] do not need to come to Addis Ababa [capital city]. Stakeholders in various areas must train women after injury to rehabilitate and live a better life. They need to be involved in the empowerment of women with the local resources they have.”

(P-P-04)

Another gap discussed by the service providers was the issue of service accessibility for women post-surgery. One provider mentioned their restricted capacity to serve only specific areas, potentially leaving women outside those regions without access to post-surgery support:

“We selected 18 intervention districts for our baseline assessment based on the information we received from the regional health bureau and the Ministry of Finance regarding the high home delivery rate. We conduct outreach activities in these areas, such as prevention, case identification, and in-kind support. Therefore, mothers outside these districts might not receive financial support. As I have told you, there are resource limitations.”

(P-P-10)

Service providers also addressed the disproportionate emphasis on surgical correction over other forms of support at fistula facilities. One psychiatrist expressed the following sentiment: “We are working below the required services for fistula patients; the main job is the surgery, and other services that need to be done are not well done.” (P-P-11).

Service providers also highlighted the fragmented care between fistula institutions and HHJ after women underwent surgical correction. A medical doctor noted the challenges of tracking women’s progress and reintegration into the community following surgery:

“After we finish [surgery], we send them to Bahirdar city [place where HHJ is located]; after that, we do not see them, and we do not know whether they returned [to their community] or stayed there [Bahirdar]…we do not have access to see what changes they have made.”

(P-P-09)

The other issue addressed by service providers is the shortage of service providers who can provide various rehabilitation services. One psychiatrist said: “We have a shortage of human power. I am the only available psychiatrist for all the patients.” (P-P-11).

In addition, another service provider discussed the issue of shortages of staffing to provide important surgical procedures post-fistula closure and noted: “We do not have doctors who can do vaginal opening surgeries, many women needed vaginal opening [due to the scar], but because we do not have a doctor, we could do nothing.” (P-P-03).

While highlighting the challenges of insufficient and fragmented services, participants also noted that bolstering economic and counseling resources is critical to comprehensive support.

#### 3.3.2. The Need for Strengthened Economic and Counseling Support

This sub-theme, the need for strengthened economic and counseling support, reveals shortcomings in formal support systems for women, as perceived by their families. While acknowledging the valuable services of fistula facilities and the HHJ, family members highlighted areas that need improvement: insufficient economic support and limited access to counseling and resources.

Nearly all the participants discussed the economic challenges women experience after completing their surgical treatment. While some family members acknowledged economic support from fistula institutions and the HHJ, they also highlighted gaps, like economic support being insufficient or not always being fulfilled as promised to women:

“Since she came home [from the fistula institutes], she has been saying she wants to work. She said I wish I had the money to work. They [HHJ] told them they would release funds, which was delayed. It [money] just came recently.”

(P-F-05)

One husband echoed the absence of promised support for women to start income-generating activities, saying:

“They told us they would give us some [money] to start a business in the form of a loan when she graduated [finalize her training]. They said they would give it to us this month, but nothing has been done since then.”

(P-F-06)

Family members discussed the vital role of economic support in facilitating women’s participation experiences, emphasizing that work is paramount for enhancing their connections with others.

“The first thing that can connect people is work, so it would be great if a job is created for them [women]…job opportunity is the way to connect to people; if a person sits at home and does nothing, [he/she] could not connect with others.”

(P-F-03)

On the other hand, one family member highlighted the sufficiency of economic support they received, yet they recommended the continuation of counseling services.

“The support is 100% enough. Their economic support is enough; they even gathered them [fistula patients] and provided support [money]. The people in Bahirdar [HHJ] provided up to 5k to initiate work, and I believe that is enough for one person. However, I still think the counseling should be continuous.”

(P-F-09)

Another participant, a daughter, also suggested psychological counseling could be beneficial for her mother, who had corrective surgery for a fistula and frequently questioned why she had to suffer from OF, saying: “My mother’s psychological condition is a bit [compromised]. I wish she could get a doctor who can counsel her…she has worries, she always asks why this thing [fistula] happened to her.” (P-F-08).

Family members underscored that women could benefit more if there were additional economic and psychological supports.

### 3.4. Nurturing Family Bonds Amidst Challenges

The theme of nurturing family bonds amidst challenges delves into how OF affects women and their families. Specifically, it reflects the critical role that family members play in supporting women suffering from this condition and the various challenges that families may encounter when serving as a support system.

#### 3.4.1. Strength in Family Unity

Family members discussed how they supported their loved ones with OF to boost their social inclusion. They provided emotional, household, and childcare support, financial aid, and help with social activities.

Many family members highlighted offering emotional support to their family members post-surgery, providing hope and encouragement for the women. For example, one participant explained how he provided emotional support for his mother, who remained incontinent despite five previous surgeries. He said: “When she loses hope, I remind her of the will of God and assure her that she is not the only one experiencing [fistula]” (P-F-O5). A husband who had undergone a leg amputation himself compared his permanent disability with his wife’s fistula and provided comfort by affirming that her condition was treatable and not hereditary. He said:

“Before she had the surgery, when she was leaking [urine], she used to be scared and sad. On the other hand, I used to encourage her and tell her that this [fistula] is not a hereditary problem…I told her I should be sad [of my disability] as it is not treatable; I told her the condition can be fixed. After that, she was happy.”

(P-F-01)

Family members also provided practical support for household chores such as cooking, cleaning, dishwashing, and childcare. A father whose wife underwent OF surgery immediately stepped in to assist with childcare post-surgery:

“When she [wife] encountered a fistula, I stayed with her for a month and a half and had her surgery at the Gondar hospital…I used to carry and watch our infant, and whenever the mother was awake, I assisted her in breastfeeding.”

(P-F-03)

Another husband underscored his support with heavy household duties to avoid the recurrence of OF. “I carry heavy loads; for example, when we run out of water, I must bring it from afar; I carry the jerrican myself.” (P-F-04).

Family members also offered financial assistance to women post-surgery, helping with household expenses and supporting their financial needs. For instance, one husband shared how he supported his wife’s income by backing her handicraft business. “She makes and sells handcrafts, such as embroidery, and uses them to buy herself clothes and shoes. As much as I can, I buy her the [materials]” (P-F-03).

Participants discussed how their extended family members provided in-kind support by giving cooking materials, grocery supplies, and shelter. For example, one husband expressed the support the couple received from the extended families of both sides:

“[Her] family supports us [providing] room, and they told us we could stay there free of charge until we build our own house; they told us to use their firewood. Moreover, we also get food and grains from my family.”

(P-F-04)

Participants highlighted how women were excluded from family and community life. One family member discussed how the woman was not participating in family events:

“The family used to look at her with confusion, and the community was from a rural area. They do not have much knowledge of fistula. So, they used to ask her what was happening, and they used to see the leakage as a joke…she would not be at her home; rather, we [I and her] would go outside the home.”

(P-F-09)

Family members preserved women’s social connections within the community by serving as representatives in places where women could not be present. One daughter underscored how she tried to maintain her mother’s social ties with her community as the mother continued to isolate herself following fistula surgery:

“Since she [her mother] will not go to [social events], I go instead …I always give excuses, saying she is sick, at work, or holy water. When you live a social life, it [participation] is a must. I am afraid that because of the isolation she experienced, she might feel a certain way. I give excuses and go to every social event; whether it is to weddings or baptisms, I miss nothing.”

(P-F 08)

During the discussion, a mother whose daughter developed OF shared how she supported her daughter by rebuilding her self-image and reconnecting her with her previous social circle after she returned from the fistula institution:

“When she came back [from fistula facilities], I made Tela [a traditional drink] and prepared food; I told the neighborhood she is cured now, and they [neighbors] were happy, and she was happy. What they say [about her] now and previously [before the treatment] is entirely different…they are being receptive.”

(P-F-07)

Despite some family members mentioning limited support from loved ones, all participants agreed on the absence of assistance from the community. This lack of support was mainly attributed to a lack of awareness about the women’s condition. One family member revealed their family’s decision to keep the OF diagnosis hidden:

“No one knew; only we and the family members knew. We kept the diagnosis a secret. Even some family members did not know she had a fistula…we only said [her diagnosis] was a simple tear, and we got that fixed; we never said she had a fistula.”

(P-F-09)

Another participant also discussed that they only received a visit from the community as a typical post-delivery tradition:

“The community knew she only gave birth; they did not know she had developed a fistula. No one offered support; of course, the visit and saying welcome home is also a great moral support. However, other than that, we have not received any form of support.”

(P-F-04)

Family members stressed their vital role in supporting women in their daily lives and fostering connections with the community despite the community’s failure to provide adequate support.

#### 3.4.2. Navigating Challenges of Family Support

Participants described that despite offering support to women with OF, family members faced significant consequences, including financial struggles, emotional challenges, and enduring negative comments and feelings of shame. Economic challenges were a common concern among family members. One husband indicated the significant loss of assets and its impact on their lives by saying: “…our life was compromised. We went through much trouble; we sold different household items that my family gave us. We had a piece of land in the countryside; we sold it and used it” (P-F 01). Another participant highlighted how his wife’s underemployment led to increased pressure on him as the primary earner for the family:

“Regarding the economy, she could not work as much as she wanted to; even if she is about to work [heavy duties], I am afraid she might experience the fistula again. Therefore, I am the only provider, and it affects me.”

(P-F-04)

Family members expressed the emotional toll of witnessing women’s suffering leading to anger, frustration, and significant distress within the family. One husband, whose wife underwent fistula surgery but still struggled from severe pain, raised concerns about her emotional state and its impact on the family dynamics saying:

“She [wife] is unhappy with her health; what can I do, or what can she do? When God is not solving the problem [pain]. We [the family] are angry because she did not get her health back or does not have a good relationship with her family.”

(P-F-03)

Despite emotional distress, husbands emphasized that their children were vital to keeping the family together. One husband said: “She has a behavior change; she is acting very angry; she has not had that kind of behavior previously…I am also living [with her] complaining because I have children.” (P-F-02).

Family members also shared the emotional burden of fistula on them, expressing ongoing concern even after the women received surgical treatment. Most participants admitted to constant worry about the possibility of fistula recurrence. One husband, whose wife’s fistula was successfully repaired but still struggled with his sexual life, highlighted concerns about their future fertility goal:

“Considering my age, even if I have a desire [sexual], I can control my [desire]. However, if there is [pain], I might think about many things. For example, would there be another illness [fistula]? How can we have another child if we want to have one?”.

(P-F-04)

On the other hand, one mother discussed her worries about fistula recurrence if her daughter got pregnant again:

“Fistula is a new thing for me. I am asking people; I ask how she can have a sexual relationship after seeing all that problem [fistula] in her life. I always tell her to divorce her husband [to prevent pregnancy].”

(P-F-07)

Family members of women with OF discussed feeling ashamed due to their loved one’s experiences with fistula. One daughter shared how her mother’s condition made her feel embarrassed about her own humanity. “It [fistula] is tough. I used to live by myself, and when I saw her [her mother] sick, I hated being a human, especially since I am a woman; it was tough.” (P-F 08).

Another participant also highlighted how his mother’s fistula was a source of shame as he grew older. “When you are a child, you do not feel anything. As you get older, you see the reaction [negative] from the community; you feel ashamed. That is how I felt after I grew older.” (P-F-05)

Despite the community’s criticism, one husband discussed how he overcame the criticism and continued to support his wife with household duties. He said:

“Some of my friends [criticized] saying, ‘You wash your wife’s clothes?’ I told them there was nothing wrong with that as she was close to me [intimate], and I told them I was the one who would have suffered had she been dead and that I did not have any problem [taking care of her].”

(P-F-01)

Participants’ experiences indicated that family members experience multidimensional consequences as support providers for women after OF surgery.

## 4. Discussion

Our study pioneered an examination of support networks for women post-OF surgery in Ethiopia. We incorporated perspectives from family members and service providers. Our analysis uncovered support types, how such supports could enhance women’s inclusion and participation, gaps in formal support, the pivotal role of families, and the challenges of families serving as support systems. Insights gained from this study can help shape better support services and policies for women with OF.

Our findings revealed the presence of formal and informal support following OF surgical repair, each offering unique and somewhat overlapping forms of assistance [48]. The formal support aimed to restore women’s well-being and foster self-sufficiency through vocational training, education, and financial aid. In Ethiopia, predominantly patriarchal society, women often rely on their husbands for support. Developing OF can result in losing this support from husbands, families, and communities, making it difficult for women to meet their basic needs compared to their counter parts. Even women who were financially independent before developing OF often lose their means of livelihood [49]. For these women, vocational and educational opportunities are crucial, as they provide the skills and support needed to participate meaningfully in society and aid their social inclusion. A report from the reintegration support program in Uganda showed women received health education, counseling, economic empowerment, advocacy, and social reintegration support after OF surgical treatment to help them resume previous responsibilities, roles, and relationships after treatment [50]. Informal support, on the other hand, assists with daily tasks, household expenses, and social relationships. Similarly, a mixed-method study from Tanzania also showed that women received support from their families with food, shelter, household chores, and provision of care for themselves and their children [51].

In this study, the formal support provided practical skills and education that opened avenues for accessing vital information and improving job opportunities. These results align with previous studies in Malawi and Tanzania, which highlighted how economic and social empowerment initiatives aid in women’s independence, dignity, and successful reintegration into their communities following OF surgery [22,52]. According to a study from Tanzania that aimed at developing a psychological intervention for women after OF, in communal societies, the central tenant of an intervention should focus on building social identities and relationships [53]. Although attempts have been made to extend formal assistance beyond surgical intervention to address the social and economic needs of women, our research findings show that the present support structures in Ethiopia fall short of meeting the financial and psychological requirements of women and their families. The constraints may be due to formal support services for women after OF surgery being provided mostly by NGOs, which are donor-based programs that have limited resources to cater to the needs of all women and their families [10,11,12,54]. To address the limited scope of donor-based support, it may be essential to integrate post-surgery fistula care into the existing healthcare and social support infrastructure, such as primary healthcare or community-based support programs. The findings of this study emphasize the necessity of strengthening formal support interventions that merge vocational training strategies with social cohesion activities for all fistula patients, to foster their social inclusion in their families and communities. Uganda’s TERREWODE reintegration support program exemplifies this approach by providing vocational and social training activities, including community-based saving and income generation. The program encourages women to join saving groups upon their return to the community and fosters solidarity through dance and drama groups, enhancing community-based support [50].

Our study revealed the importance of formal support that acknowledges and incorporates women’s spiritual beliefs. Previous research has shown that women with fistula often face exclusion from religious services [55,56] and struggle to come to terms with why they have suffered such a tragic fate. In Ethiopia, where cultural and religious practices are deeply woven into society, integrating acceptable spiritual practices into formal support systems could help address both medical and spiritual needs to enable women to reconcile their conditions, which have previously been seen as a punishment or incurable affliction [57,58]. Future research is needed to understand the role of spirituality and spiritual practices as a support system for women.

Our study underscores the vital role of family members as the primary source of natural support for women post-OF surgery, highlighting the profound impact of OF on caregivers. Past research revealed divergent experiences regarding family and informal support availability among affected women. For example, studies from Nigeria reported a concerning lack of familial support for women with OF, persisting even after surgery [36,59,60], while one study from Tanzania [51] and one study from Kenya [61] each reported receiving tangible, financial, and emotional support from at least one family member post-surgery.

In systems like Ethiopia’s, where formal structures are limited, reliance on natural support persists, yet women with OF often remain detached from such support, as noted in previous qualitative studies from Ethiopia [62,63]. Our study showed that when family support still exists for women with OF, it often comes with emotional and economic challenges. This echoes the findings of a critical ethnography study in Ghana, which found the emotional burden experienced by caregivers within families, including feelings of sadness, uncertainty, shame, remorse, and grief, often stemming from past experiences with OF and fears of its recurrence [64].

We also found that husbands typically provided informal support to women post-OF surgery when they were not divorced. This contrasts with the exploratory qualitative study from Kenya, which found that women often receive care from their natal families rather than their marital ones [61]. However, the support from husbands has been found in other studies from Ghana and Kenya to be frequently accompanied by issues such as a lack of intimacy and concerns about infertility, leading most likely to strained marital relationships [64,65].

Enhancing sustainable and culturally appropriate support networks could significantly improve the social inclusion and participation of women post-OF surgery. Addressing the challenges within existing natural support systems can lead to better outcomes for these women. Additionally, future research should consider exploring potential sources of informal support beyond the immediate family, such as friends or neighbors.

### Limitations of This Study

This study has limitations. First, family participants were selected based on their relationship with women who had OF surgery, potentially biasing it towards more supportive members. Thus, the findings may not apply universally to all post-OF surgery women. Second, only service providers from certain institutions and NGOs were included, possibly limiting findings to support within these organizations. Lastly, translation from Amharic to English may have resulted in some loss of meaning; we aimed to mitigate this by postponing translation until theme development [66]. The findings of this study are not generalizable to other locations due to the inherent limitations of qualitative research, such as the participant selection process and the small sample size. We acknowledge that sociocultural contexts and perceptions of OF can vary widely across different settings.

## 5. Conclusions

This study’s findings suggest that while efforts are being made to provide comprehensive support for women with OF post-surgery, there are still gaps in addressing the complex physical, psychological, and social challenges faced by women. Instead of relying solely on donor-based support at specific locations, a collaborative effort between the government and NGOs is essential. Integrating support into the established system can enhance the accessibility of formal support services, making them more readily available to women. Providing services directly to families of women with OF could also enable these families to provide long-term, sustainable support for women. Future research should explore available formal support services within the community beyond the support given at the fistula facilities and the existing social networks beyond the immediate family members and how these networks could contribute to women’s social inclusion and participation experiences after OF surgery.

## Figures and Tables

**Table 1:** Participant demographics.

Characteristics	Service Providers (N = 11)	%
**Level of education**		
BSc	1	9.0
MSc and above	10	90.9
**Profession**		
Nurse	3	27.2
Medical doctor	1	9.0
Spiritual therapist	1	9.0
Psychiatrist	2	18.1
Psychotherapist	1	9.0
Physiotherapist	1	9.0
Social worker	1	9.0
Public health	1	9.0
**Years of service**		
<5	2	18.1
>5	9	72.7
**Characteristics**	**Family**	**%**
**Age**		
20–24	1	11.1
25–29	3	33.3
30–34	1	11.1
35–39	2	22.2
>40	2	22.2
**Gender**		
Male	6	66.6
Female	3	33.3
**Residence**		
Urban	3	33.3
Rural	6	66.6
**Relationship to the women**		
Child	2	22.2
Parent	1	11.1
Spouse	5	55.5
Cousin	1	11.1
**Occupation**		
Daily laborer	2	22.2
Self-employed	2	22.2
Farmer	2	22.2
None	3	33.3
**OF status of women after surgery**		
Continent	8	88.8
Incontinent	1	11.1

**Table 2:** Themes and sub-themes.

Theme	Sub-Theme
Holistic support provision	Vocational and educational opportunities
	Spiritual, psychological, and physical support
Targeted interventions and supports to enhance social participation	Rebuilding connections with peers and family
	Nurturing dignity through family-like care
Unveiling gaps in the formal support system	Insufficient and fragmented services
	The need for strengthened economic and counseling support
Nurturing family bonds amidst challenges	Strength in family unity
	Navigating challenges of family support

## Data Availability

Data are available by a reasonable request to the corresponding author.
